# Oxidized LDL promotes EMS-induced angiogenesis by increasing VEGF-A expression and secretion by endometrial cells

**DOI:** 10.1186/s10020-022-00582-6

**Published:** 2022-12-12

**Authors:** Caiqi Ma, Wei Huang, Hui Wang, Wenxia Yao, Min Liang, Guifang Yu, Xinke Zhou

**Affiliations:** 1 Department of Oncology, Key Laboratory of Biological Targeting Diagnosis, Therapy and Rehabilitation of Guangdong Higher Education Institutes, The Fifth Affiliated Hospital of Guangzhou Medical University, No. 621, Gangwan Road, Huangpu District, Guangzhou, 510530 China; 2 Reproductive Medical Center, Affiliated Guangzhou Women and Children’s Medical Center of Guangzhou Medical University, No. 9, Jinsui Road, Guangzhou, 510530 China

**Keywords:** Endometriosis, Vascular metastasis, Angiogenesis, OxLDL, VEGF-A, AKT-HIF-1α signaling

## Abstract

**Background:**

Endometriosis (EMS) is a “tumour-like” gynaecological disease with distant metastasis, and studies have shown that EMS can induce distant metastasis through vascular vessels, but the driving factors and their mechanism are not clear.

**Methods:**

We used an EMS animal model and gene knockout technique to explore the role of EMS-induced angiogenesis in EMS metastasis in vivo and in vitro and clarify the role and molecular mechanism of oxLDL in promoting EMS-induced angiogenesis.

**Results:**

We found that microvascular density (MVD) in metastasized ectopic endometrium and eutopic endometrial tissue was higher than that in normal endometrial tissue, and plasma oxLDL was positively correlated with the distant metastasis of EMS. Furthermore, we clarified that oxLDL enhanced the MVD of endometrial tissue by increasing VEGF-A expression and secretion in endometrial cells. Finally, we illustrated the mechanism by which oxLDL promotes VEGF-A expression through the AKT-HIF-1α signalling pathway.

**Conclusion:**

OxLDL is a risk factor promoting distant EMS metastasis by increasing VEGF-A expression and secretion through AKT-HIF-1α signalling. This finding may provide theoretical support and therapeutic targets for the clinical prevention and treatment of EMS.

**Supplementary Information:**

The online version contains supplementary material available at 10.1186/s10020-022-00582-6.

## Introduction

Endometriosis (EMS) is a gynaecologic disease with high incidence and a lack of radical cure (Zondervan et al. 2020). It not only seriously affects the physical and mental health of women but is also one of the main causes of infertility (Zondervan et al. 2018; Evans and Decherney 2017). The aetiology of EMS is complex, and its pathogenesis has not been fully clarified (Horne and Saunders 2019). EMS is difficult to cure due to its ability to metastasize, including implant metastasis from the fallopian tube, lymphatic metastasis and vascular metastasis; among them, vascular metastasis is the predominant metastasis (Samani et al. 2019; Machado et al. 2008; Keichel et al. 2011). Unfortunately, the specific mechanism is still unclear. Studies have shown that the microvessels in endometrial tissue are related to the occurrence and development of EMS, and endometrial cells may metastasize remotely through blood vessels (Laschke and Menger 2018; Laschke et al. 2011). However, the factors that promote the metastasis of endometrial cells still require further elucidation. Low-density lipoprotein (LDL) is a kind of lipoprotein that carries cholesterol into peripheral tissue cells. LDL can be oxidized into oxidized low-density lipoprotein (oxLDL), which easily causes arteriosclerosis by accumulating on the walls of arteries and veins (Pirillo et al. 2013). New studies have reported that oxLDL is also closely related to the occurrence and development of tumours (Ma et al. 2019; Yang et al. 2021). Recent studies found that oxLDL is correlated with the progression of EMS (Polak et al. 2011; Polak et al. 2013). However, the molecular mechanism requires deeper investigation.

In the process of vascular metastasis of tumour cells, tumour cells secrete proangiogenic factors to induce angiogenesis so that they can enter the blood vessels for distant metastasis (Yang et al. 2013). Additionally, oxLDL can enhance tumour-induced angiogenesis to promote the vascular metastasis of cancer cells (Han et al. 2021), indicating that oxLDL may promote EMS-induced angiogenesis to assist EMS vascular metastasis. Tumour angiogenesis requires vascular epithelial factors such as VEGF-A, VEGF-B and PDGF to initiate the proliferation and migration of vascular epithelial cells (Claesson-Welsh and Welsh 2013; Saharinen et al. 2011). For EMS, the vascular epithelial factor that is involved in EMS-induced angiogenesis remains unknown. In this study, we attempted to explore the role of EMS-induced angiogenesis in EMS vascular metastasis and clarify the molecular mechanism by which oxLDL promotes EMS-induced angiogenesis.

## Materials and methods

### Cell lines and cell culture

Primary hEECs, hESCs and HUVECs were purchased from Procell Biotech (Wuhan, China). All cells were maintained in specific medium with 10% (v/v) FBS, 100 U/ml penicillin, and 100 mg/ml streptomycin and incubated at 37 °C in a humidified incubator at 5% CO_2_.

### Tissue specimens and clinicopathological characteristics

Normal endometria were obtained from uterine curettage endometria that were tested normally, while eutopic endometria and ectopic endometria were obtained from EMS patients. The age of all volunteers was between 20 and 40 years old. The patients in the control group underwent diagnostic curettage in a physical examination, and their pathological results were normal. In the EMS group, patients with pathologically confirmed EMS were selected. The samples were collected and histopathologically and clinically diagnosed at the Fifth Affiliated Hospital of Guangzhou Medical University between 2020 and 2021. Written informed consent was obtained from all patients prior to the study. The use of the clinical specimens for research purposes was approved by the Institutional Research Ethics Committee.

### Experimental animals

Mature (13–15 weeks old) Wistar rats were purchased from Guangdong Medical Laboratory Animal Center (Guangzhou, China). Rats were allowed to acclimate to local conditions for at least 1 week and maintained under a 12 h dark, 12 h light cycle with food and water ad libitum. The care, use, and treatment of all animals in the present study were in strict agreement with the institutionally approved protocol according to the United States Public Health Service (USPHS) Guide for the Care and Use of Laboratory Animals, as well as the guidelines set forth in the Care and Use of Laboratory Animals by Guangzhou Medical University. The animal use protocol was reviewed and approved by the institutional animal care and use committee of Guangzhou Medical University.

### Establishment of the EMS rat model

Mature (13–15 weeks old) Wistar rats were used to establish the EMS animal model. After intraperitoneal anaesthesia with 10% chloral hydrate (0.38 ml/100 g body weight), a longitudinal incision with a length of 2–3 cm was made approximately 1 cm above the pubic symphysis, a “Y”-shaped uterus was found on the dorsal side of the bladder, the morphology of the uterus was observed to keep the uterus in a relaxed position, and the diameter of the uterus was measured with a Vernier calliper. Approximately 1.5 cm from the bifurcation of the uterus, the first ligation line was ligated with silk thread, the second ligation line was ligated near the ovarian end, and the uterus approximately 1.5 cm in between was intercepted. The uterus was cut from the first ligation line, and the uterine segment was cut longitudinally. Then, the endometrial layer and serosa layer were torn with small tweezers, and the serosa layer was repaired. Endometrial segments with a length, width and height of approximately 5 mm were trimmed. The four corners of the trimmed endometrial segments were sutured with a nondestructive suture on the right abdominal wall, and then, the abdomen was closed. Part of the remaining endometrial fragments was embedded in paraffin to confirm that the transplanted tissue was endometrium and left for subsequent experiments. After the operation, the rats were kept for 15 days, and then, the modelling effect was confirmed. The ectopic scar of the rats that were confirmed to be successful in modelling (obvious ectopic scar was observed) was dissected and separated. After paraffin embedding, staining was performed. After the ectopic endometrial tissue was confirmed by microscopic observation, IHC detected CD34, the marker of vascular endothelial cells, and the vascular density was quantified. The vascular density and lumen size of the endometrium were compared between the model group and the normal control group (sham operation group).

### Tube formation assay

The human umbilical vein endothelial cell (HUVEC) in vitro tube formation assay was performed by first pipetting 200 µl of Matrigel (BD Biosciences, Franklin Lakes, NJ, USA) into each well of a 24‑well plate, followed by polymerization for 30 min at 37 °C. HUVECs (2 × 10^4^ cells; Procell Life Technology Co., Ltd., Wuhan, China) in 200 µl of conditioned medium (culture medium from hEECs and hESCs treated with oxLDL) were added to each well and incubated at 37 °C in an atmosphere containing 5% CO_2_ for 12 h. Images were captured using a bright field with a Zeiss Axio Observer Z1 (Carl Zeiss AG, Oberkochen, Germany).

### RNA-seq

RNA sequencing was performed with the Illumina platform. In brief, the first step in the workflow involved purifying the poly-A-containing mRNA molecules using poly-T oligo-attached magnetic beads. Following purification, the mRNA was fragmented into small pieces using divalent cations under elevated temperature. The cleaved RNA fragments were copied into first-strand cDNA using reverse transcriptase and random primers. This step was followed by second-strand cDNA synthesis using DNA Polymerase I and RNase H. These cDNA fragments then had the addition of a single ‘A’ base and subsequent ligation of the adapter. The products were then purified and enriched with PCR amplification. We then quantified the PCR yield by Qubit and pooled samples together to make a single strand DNA circle (ssDNA circle), which yielded the final library. DNA nanoballs (DNBs) were generated with the ssDNA circle by rolling circle replication (RCR) to enlarge the fluorescent signals during the sequencing process. The DNBs were loaded into the patterned nanoarrays, and single-end reads of 50 bp were read through the Illumina platform for the following data analysis study. For this step, the Illumina platform combined the DNA nanoball-based nanoarrays and stepwise sequencing using the Combinational Probe-Anchor Synthesis Sequencing Method. Tools such as bcl2fastq, FastQC, and hisat2 were used for the following bioinformatics analysis. In the analysis of differentially expressed genes (DEGs), the genes that had more than a twofold change and a corrected P value less than or equal to 0.05 were defined as DEGs. With DEGs, we performed KEGG pathway classification and gene set enrichment analysis (GSEA) using R. We uploaded the sequence data into the online database SRA (Sequence Read Archieve). Our sequence data are numbered JD-KYFW-2022-874-JSFW-01.

### Bioinformatics analysis

Gene set enrichment analysis (http://software.broadinstitute.org/gsea/msigdb/index.jsp) software programs were used to analyse the EMS Genome Atlas.

### Enzyme-linked immunosorbent assay

The supernatant VEGF-A of hEECs treated with different concentrations of oxLDL was detected by a VEGF-A ELISA kit (catalogue number: RAB0507, MERCK, Germany) following the procedure from the manufacturer. The culture medium was obtained after the hEECs were treated with oxLDL for 24 h. The plasma oxLDL of the EMS patients and the controls was tested with freshly collected plasma from the EMS patients and healthy controls, which were frozen and stored in liquid nitrogen before use. The samples were collected and histopathologically and clinically diagnosed at the the Fifth Affiliated Hospital of Guangzhou Medical University between 2020 and 2021.

### H&E staining and immunohistochemistry

Tissues were surgically resected, fixed in formalin and embedded in paraffin. Then, 4.0 mm thick histologic sections were prepared. Before immunohistochemistry, histologic sections were stained with haematoxylin–eosin to observe the morphology. The sections were treated with endogenous peroxidase blocking solution and normal goat serum to block nonspecific background. The sections were then incubated with antibodies against CD34 and VEGF-A (CST, Danvers, USA). After overnight incubation at 4 °C, the sections were incubated with a biotin-conjugated secondary antibody at room temperature for 20 min and were incubated with enzyme conjugate (HRP-Streptavidin) under the same conditions. The vessels were revealed with streptavidin-peroxidase followed by the chromogenic substrate diaminobenzidine (DAB), and the sections were counterstained with haematoxylin. Images of tissue microarrays were taken by Pannoramic Viewer software, and quantification of the expression level was determined by greyscale scanning with ImageJ software.

The microvessel density (MVD) was performed by immunostaining using endothelial marker anti-CD34. The whole endometrium section was scanned at low power by microscope and identified the area of highest MVD. Then individual microvessels were counted at higher power (X200 field) in an adequate area. The MVD of the endometrial tissues were determined by counting CD34-positive areas in 10 fields/serial sections from 5 tissues per group. Any stained endothelial cell or clusters separated from adjacent vessels were counted as a single microvessel, even in the absence of vessel lumen. Every single count was expressed as the highest number of microvessels identified at the area. Negative controls were incubated without the primary antibody.

### Western blotting

HIF-1α and p-AKT antibodies were purchased from Abcam (Cambridge, Massachusetts, USA), and OLR1 was purchased from Santa Cruz Biotechnology (Santa Cruz, CA, USA). VEGF-A, GAPDH and β-actin antibodies were purchased from CST (Danvers, MA, USA). Cells were harvested and lysed for total protein extraction. The protein concentration was determined using a BCA protein assay kit (Keygen, Nanjing, China) according to the manufacturer's protocol. Aliquots of equal amounts of protein from the cell lysate were subjected to Western blot analysis. Densitometry was performed using ImageJ software with normalization to β-actin levels.

### siRNA transfection

OLR1 siRNA and a nonspecific siRNA (control) were purchased from RiboBio (Guangzhou, Guangdong, China). According to the manufacturer’s instructions, transfections were performed at approximately 60% confluency using Lipofectamine 2000 (Invitrogen, Carlsbad, CA). For each transfection reaction, 20 nM OLR1 siRNA or control siRNA was used for preparation of siRNA-transfection complexes at room temperature for 20 min. Transfections were performed in 0.5 (12-well plate) or 1.5 mL (6-well plate) serum-free medium for 8 h. After incubation, transfection complexes were removed and replaced with their corresponding media. Transfection efficiency (80–90%) was determined by Western blotting analysis. Cells were utilized for other experiments at 24–72 h after transfection.

### Immunofluorescence

hEECs were plated on culture slides and incubated with siRNA and/or oxLDL (50 µg/ml) for 12 h. The cells were washed and fixed in 4% paraformaldehyde. Then, the cells were blocked with normal nonimmune goat serum at 37 °C for 1 h. After three washes, they were incubated with rabbit phospho-Akt and HIF-1α antibodies (Abcam, Cambridge, USA) at 37 °C for 2 h and then incubated with FITC-conjugated goat anti-rabbit IgG (Dako, Glostrup, Denmark) at 37 °C for 1 h after three washes. Finally, the cell nucleus was stained with 4,6-diamino-2-phenylindole (DAPI) (Sigma). Cells were visualized under a confocal microscope (Carl Zeiss, Oberkochen, Germany). In negative control staining, the primary antibodies were omitted.

### Statistical analysis

Data are presented as the means ± SDs. Comparisons were performed by two-tailed paired Student’s t tests. A value of p < 0.05 was considered statistically significant.

## Results

### EMS induced angiogenesis of the endometrium

**Fig. 1:**
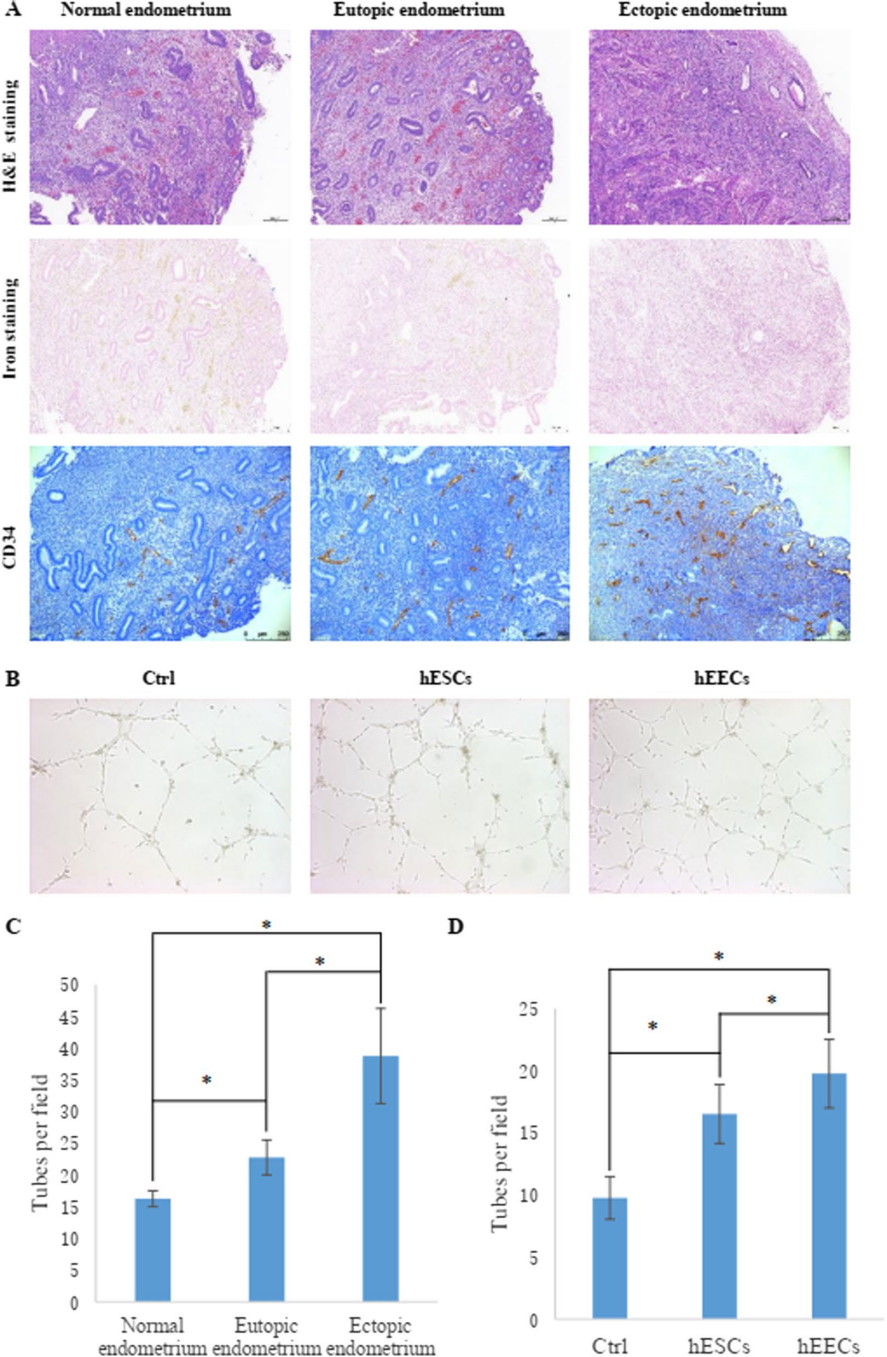
EMS induced angiogenesis in endometrial tissue. **A** The microvessel density in normal endometrium, eutopic endometrium and ectopic endometrium. The microvasculature was stained for CD34 by immunohistochemistry, while all the tissues were also stained by H&E and iron. The number in each group was 8. **B** Tube formation of hUVECs induced by hESCs and hEECs in a coculture system with chambers. **C** Histogram representing the microvessel density in normal endometrium, eutopic endometrium and ectopic endometrium. The number in each group was 8. **D** Histogram representing the tube number in each group. The number in each group was 3. * represents p < 0.05

Distant metastasis similar to that of tumour cells is one of the important reasons why EMS is difficult to cure. Among the types of metastasis, vascular metastasis is important, and angiogenesis is an indispensable basis for vascular metastasis. Microvessel density (MVD) is a good indicator of angiogenic activity. We used the vascular endothelial cell-specific marker CD34 to immunohistochemically mark the microvessels of endometrial tissue and then analysed the difference in microvessel density between normal endometrial tissue and EMS eutopic and ectopic endometrial tissues. The results showed that the endometrial microvessel density in EMS endometrial tissue was higher than that in the healthy control group, and the density in EMS ectopic endometrial tissues was higher than that in eutopic endometrial tissues (Fig. 1A, C). Then, we isolated human endometrial epithelial cells (hEECs) and endometrial stromal cells (hESCs) from normal endometrial tissue and incubated human umbilical vein endothelial cells (HUVECs) laid on Matrigel with their culture medium. Then, their effects on the tube formation of HUVECs were observed through a tube formation assay. The results showed that both hEECs and hESCs secreted factors to enhance the tube formation of HUVECs (Fig. 1B, D). These outcomes indicated that endometrial cells can induce angiogenesis in the endometrium.

### OxLDL enhanced the angiogenesis of EMS endometrial tissue

**Fig. 2:**
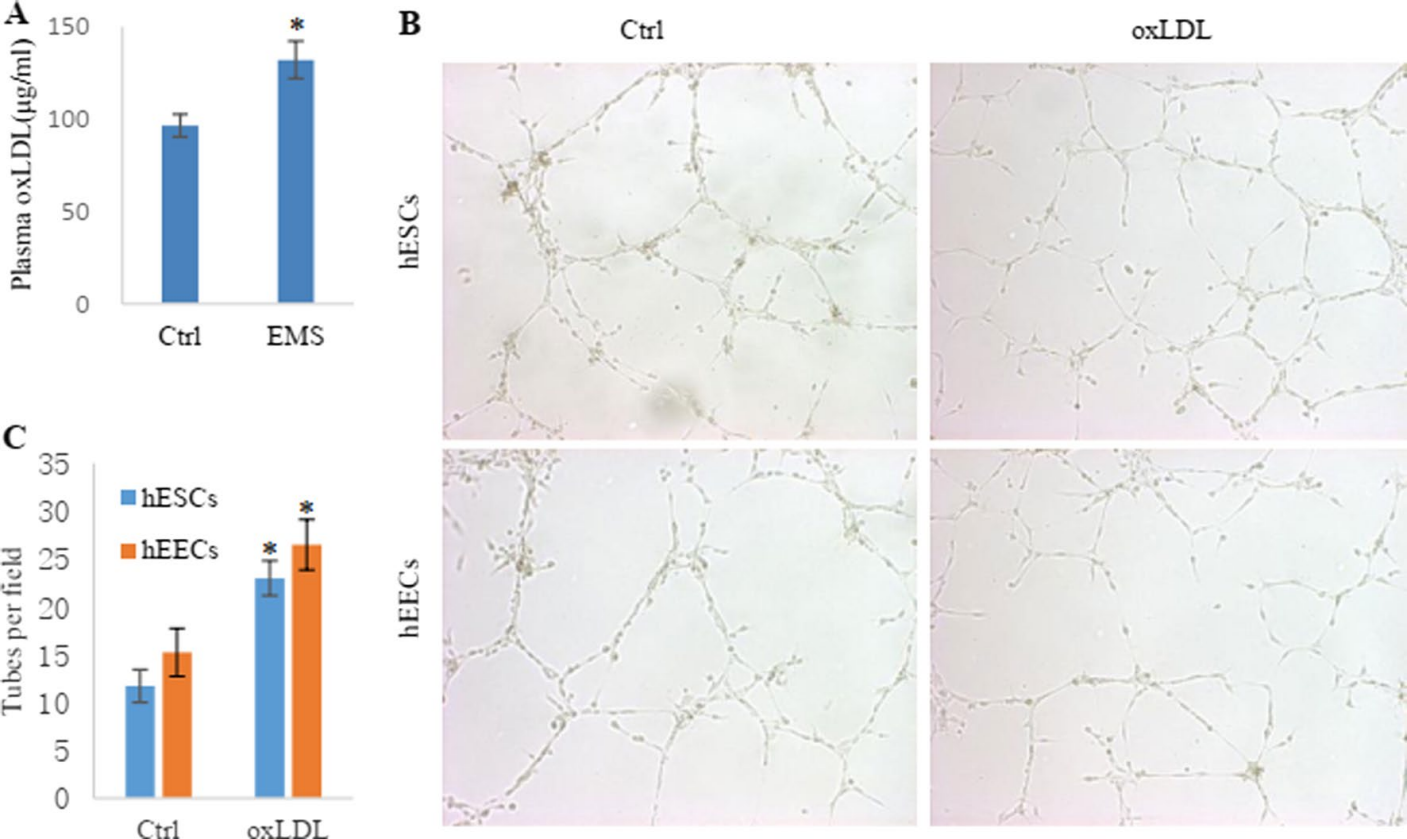
OxLDL promoted EMS-induced angiogenesis. **A** The plasma oxLDL level was elevated in EMS patients. The number in each patient group was 8. **B**, **C** Tube formation induced by hESCs and hEECs treated with oxLDL and PBS. The histogram represents the tube number in each group. * represents p < 0.05

**Fig. 3:**
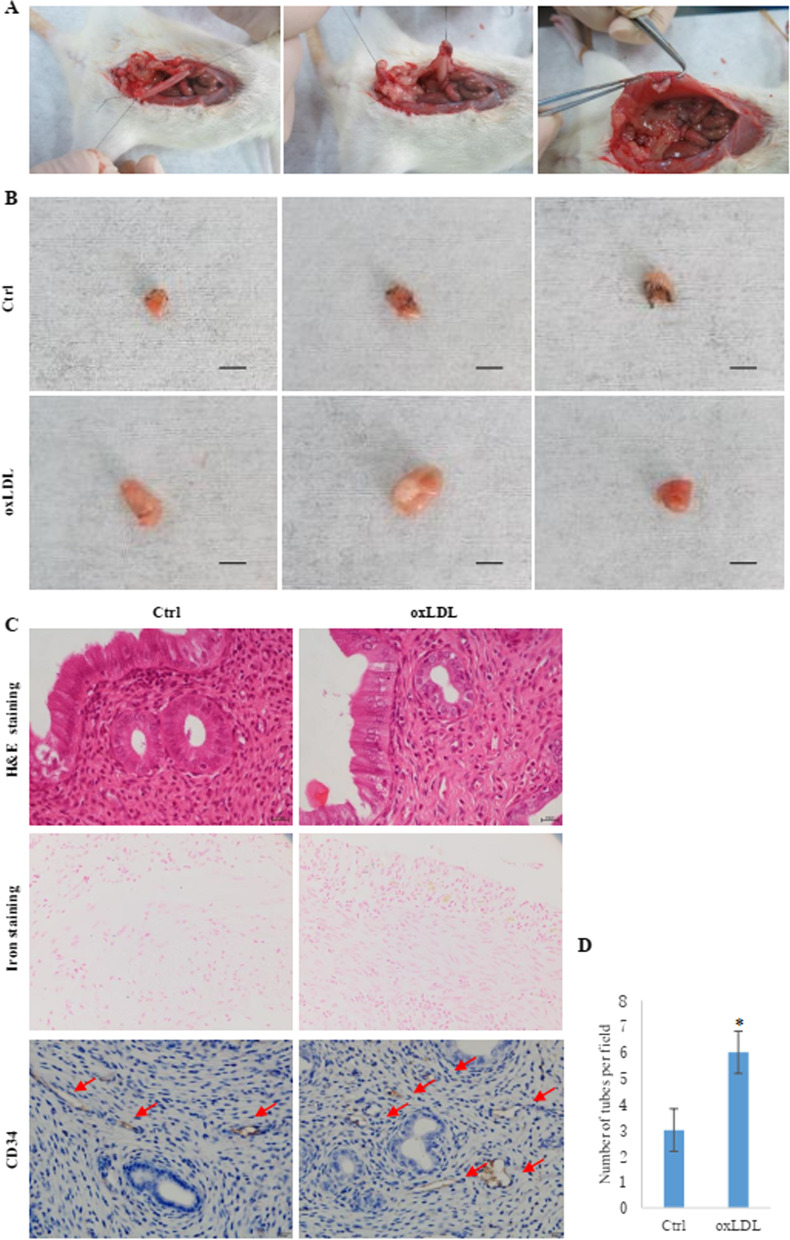
OxLDL promoted the growth of ectopic endometrium in EMS animal models. **A** Three steps for establishment of the EMS animal model. **B** The ectopic endometrium in each group of EMS animal models. The scale bar represents 5 mm. (**C**, **D**) MVD in the ectopic endometrium from the two groups of animal models. The microvasculature was stained for CD34 by immunohistochemistry, while all the tissues were also stained by H&E and iron. The MVD was represented by the number of tubes in each field, and the numbers were counted with ImageJ software. The histogram represents the MVD in each group. The number in each group was 5

To explore the factors that cause abnormal enhancement of angiogenesis in EMS endometrial tissue, we collected the plasma of healthy controls and EMS patients (Table 1) and detected the concentration of oxLDL with an ELISA kit. It was found that the plasma concentration of oxLDL in the EMS patients was higher than that in the healthy controls (Fig. 2A). To further explore whether the abnormally increased oxLDL affected the angiogenesis of endometrial tissues in EMS patients, we incubated the hEECs and hESCs with oxLDL and then applied the supernatants to culture the HUVECs to observe the effect on tube formation. The results showed that oxLDL enhanced the ability of hEECs and hESCs to induce tube formation (Fig. 2B, C). For a deeper investigation to demonstrate the effect of oxLDL on vascular metastasis of EMS in vivo, we established an EMS rat model (Fig. 3A). After the EMS rat models were successfully established, oxLDL and PBS were used to continuously administer drugs to the two groups of rats for 30 days, and then, the ectopic endometrial tissues were removed by surgery for analysis. The results showed that the endometrial tissue of the oxLDL-treated group grew larger than that of the control group (Fig. 3B). Using CD34 to mark the microvessels in the endometrial tissues, we found that the MVD in the oxLDL-treated group was higher than that in the control group (Fig. 3C, D). These results suggest that oxLDL can promote angiogenesis in the endometria of rats with EMS.

**Table 1:** Clinical characteristics of the plasma samples

Characteristic	Ctrl	EMS	*p* value
Number	8	8	
Age (years)	35.25	36.13	0.168
BMI	21.25	22.18	0.055
TC (mg/dL)	2.49	2.58	0.052
LDL-C (mg/dL)	1.37	1.43	0.021
oxLDL (μg/dL)	99.63	132.14	0.007

*BMI* body mass index, *TC* total cholesterol, *LDL-C* low-density lipoprotein cholesterol. Statistical significance was determined by the Chi-square test

### OxLDL upregulated the expression and secretion of VEGF-A in endometrial cells

**Fig. 4:**
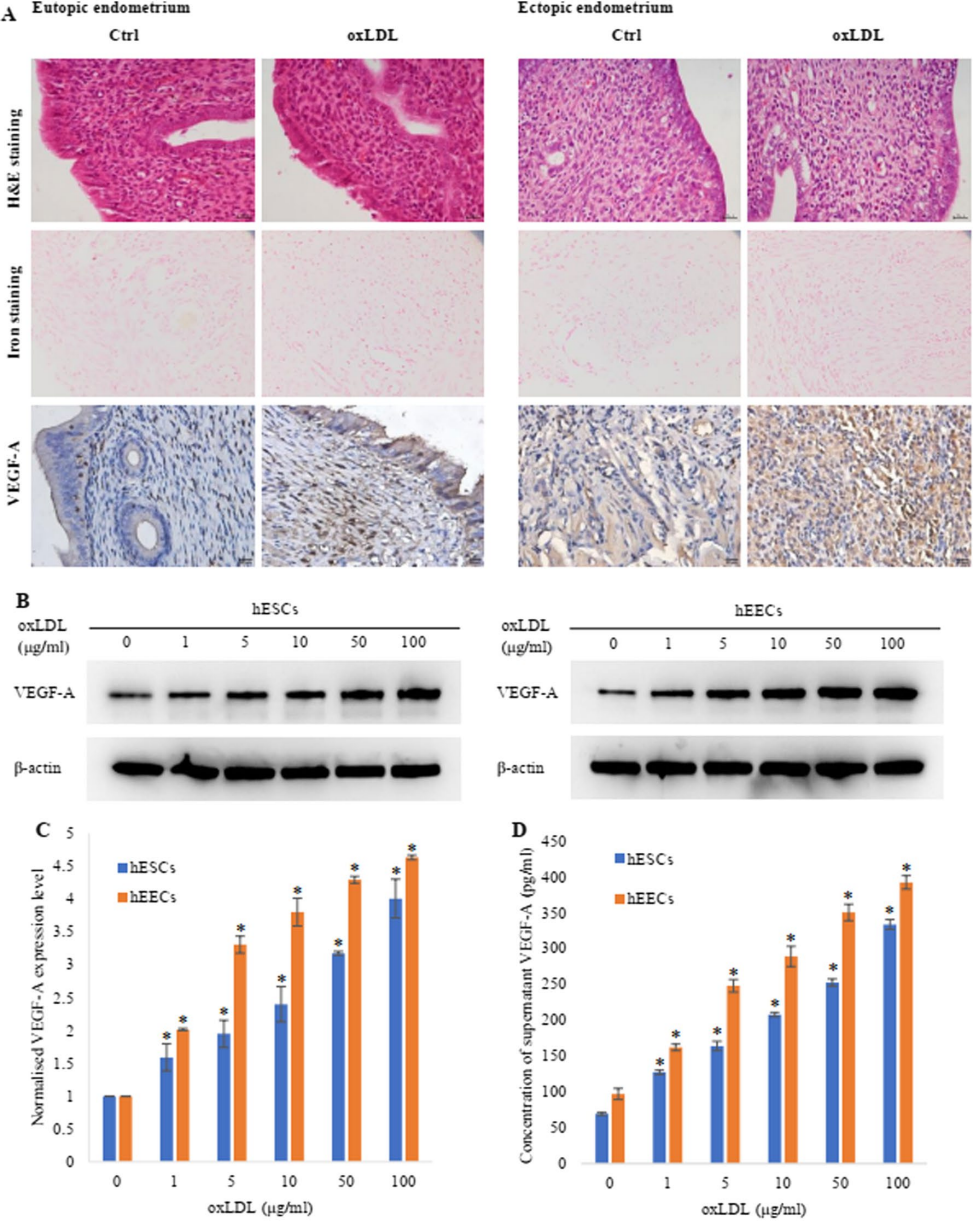
OxLDL increased the expression and secretion of VEGF-A in endometrial cells. **A** VEGF-A expression in eutopic endometrium and ectopic endometrium from the animal models treated with oxLDL (dissolved in PBS solution) or the same volume of PBS for 48 h. VEGF-A was stained by immunohistochemistry, while all the tissues were also stained by H&E and iron. **B**, **C** Western blot analysis of VEGF-A expression in the hESCs and hEECs treated with oxLDL and PBS. The histogram represents the greyscale of each lane from the Western blot. The number in each group was 3. * compared with the 0 µg/ml oxLDL group, p < 0.05. **D** The VEGF-A secretion of the hESCs and hEECs treated with oxLDL and PBS for 48 h was tested by ELISAs. The number in each group was 3. * compared with the 0 µg/ml oxLDL group, p < 0.05

Vascular epithelial growth factor A (VEGF-A) is an important factor in promoting angiogenesis. To explore the mechanism by which oxLDL promotes endometrial angiogenesis, we detected the expression level of VEGF-A in endometrial cells of the EMS rat model by immunohistochemical techniques. The expression level of VEGF-A in endometrial cells of the oxLDL-treated rats was higher than that in the control rats (Fig. 4A), indicating that oxLDL can upregulate the expression of VEGF-A in EMS endometrial cells. Furthermore, we detected the expression of VEGF-A in hEECs and hESCs after oxLDL treatment by Western blotting and found that the expression of VEGF-A in both hEECs and hESCs treated with oxLDL increased significantly, especially in hESCs (Fig. 4B, C). Finally, ELISAs were used to detect the concentration of VEGF-A in the culture medium of hEECs and hESCs after oxLDL incubation. We found that the concentration of VEGF-A in the oxLDL incubation group was higher (Fig. 4D), suggesting that oxLDL can enhance the secretion of VEGF-A in hEECs.

### OxLDL upregulated VEGF-A expression in endometrial epithelial cells via the PI3K-AKT-HIF-1α signalling pathway

**Fig. 5:**
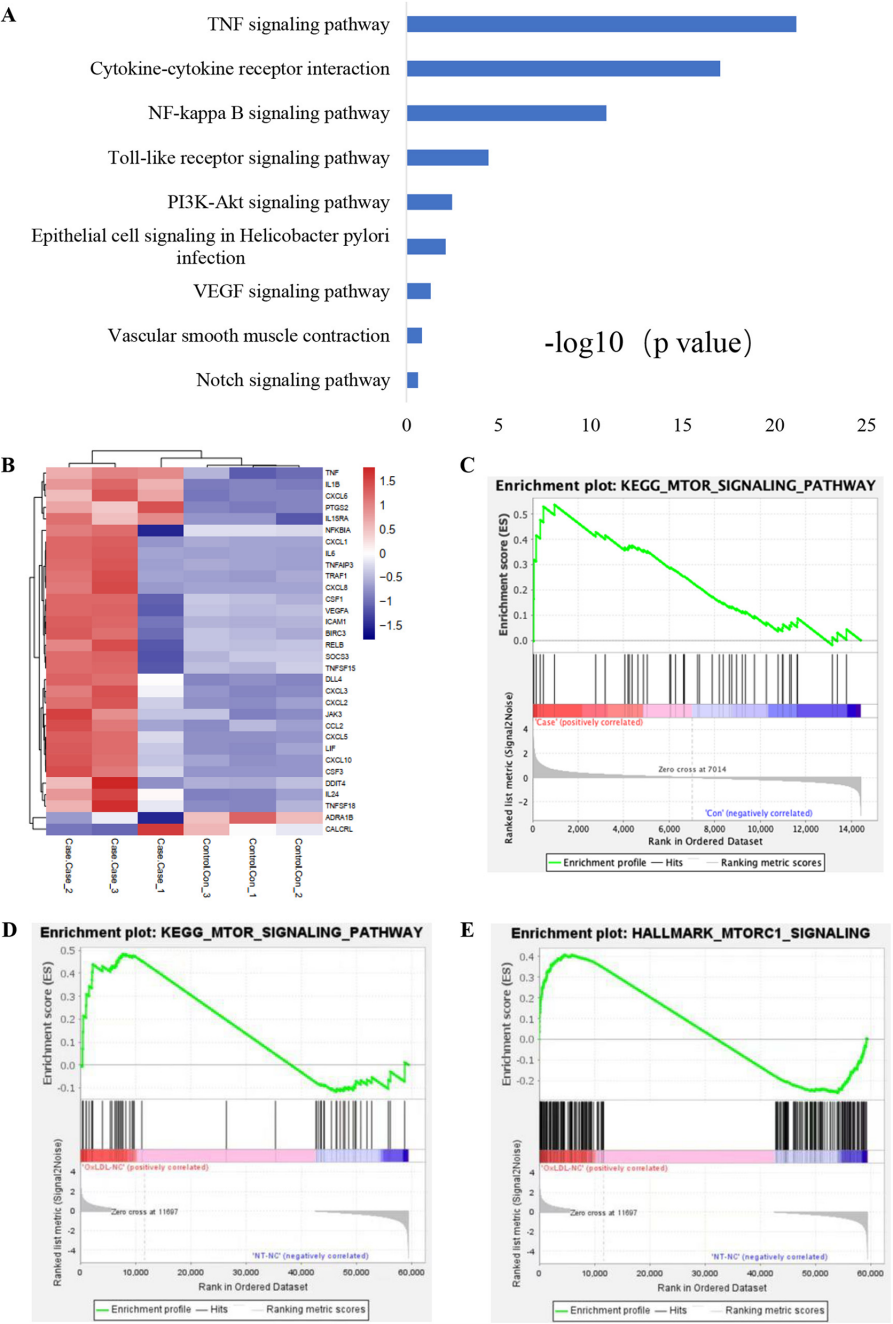
RNA-seq analysis predicted the potential mechanism by which oxLDL upregulates VEGF-A expression. **A** KEGG analysis of the hEECs treated with oxLDL and PBS showed the possible signalling pathway involved in the process of oxLDL upregulating VEGF-A. **B** Heatmap analysis predicted that VEGF-A may be upregulated by oxLDL. **C**–**E** The GSEA plot shows that VEGF-A expression positively correlates with AKT-mTOR-activated gene signatures (MA_ENDOMETRIAL_EPITHELIAL_CELL, MINTA_VASCULAR_SMOOTH_MUSCLE_CELL, YANG_BLADDER_CANCER_CELL)

**Fig. 6:**
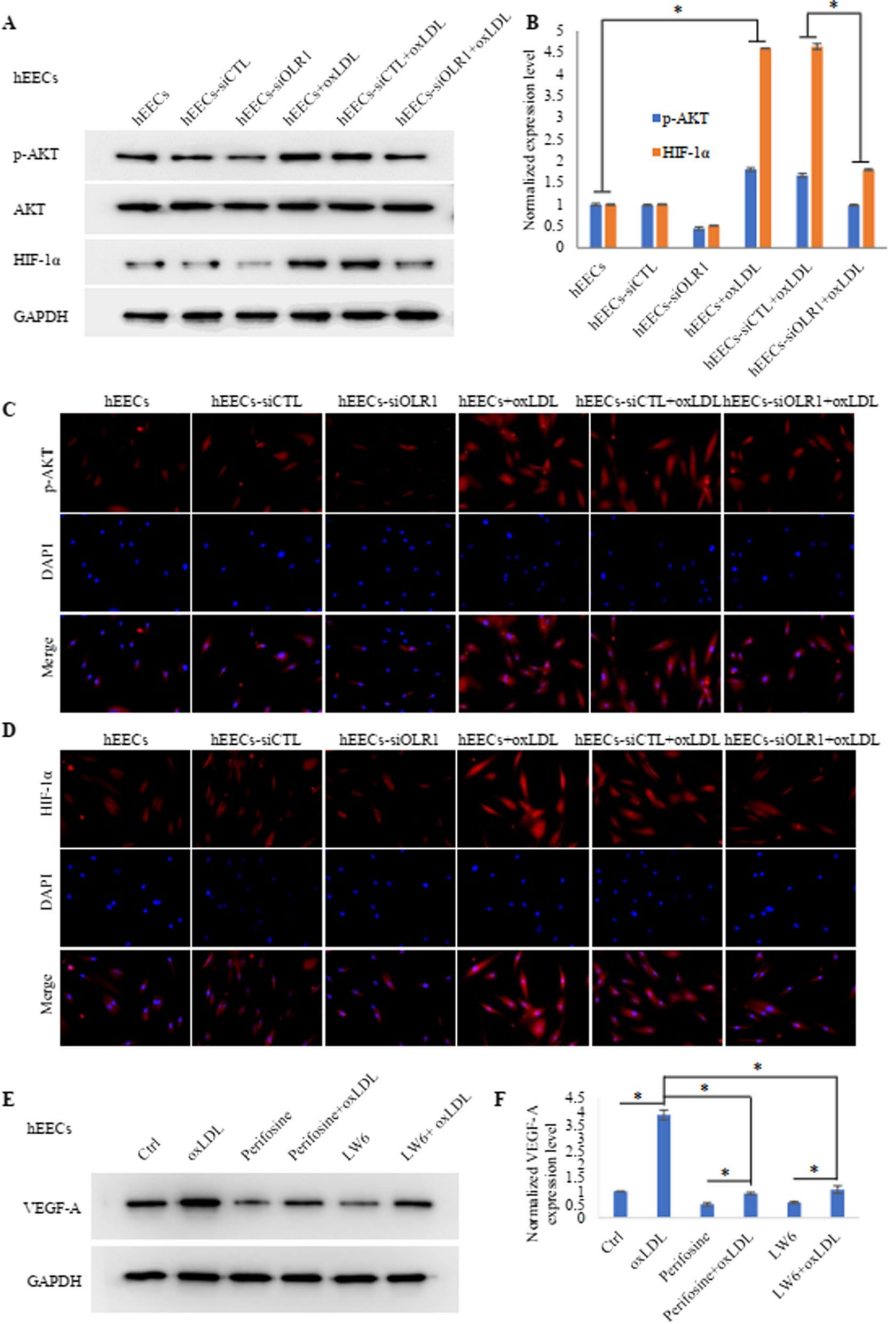
OxLDL upregulated VEGF-A expression and secretion via OLR-1/PI3K/Akt signalling. **A**, **B** p-AKT and HIF-1α expression in each group of hEECs treated with oxLDL (50 μg/ml) or PBS for 48 h was examined by Western blots. The histogram represents the greyscale of each lane from the Western blot. The number in each group was 3. * represents p < 0.05. **C**, **D** p-AKT and HIF-1α expression in the hEECs treated with oxLDL (50 μg/ml) or PBS for 48 h was examined by immunofluorescence. **E**, **F** VEGF-A expression in each group of hEECs treated with oxLDL (50 μg/ml) for 48 h, the AKT inhibitor perifosine (20 mM) and HIF-1α LW6 (20 mM) was examined by Western blotting. The histogram represents the greyscale of each lane from the Western blot. The number in each group was 3. *represents p < 0.05

**Fig. 7:**
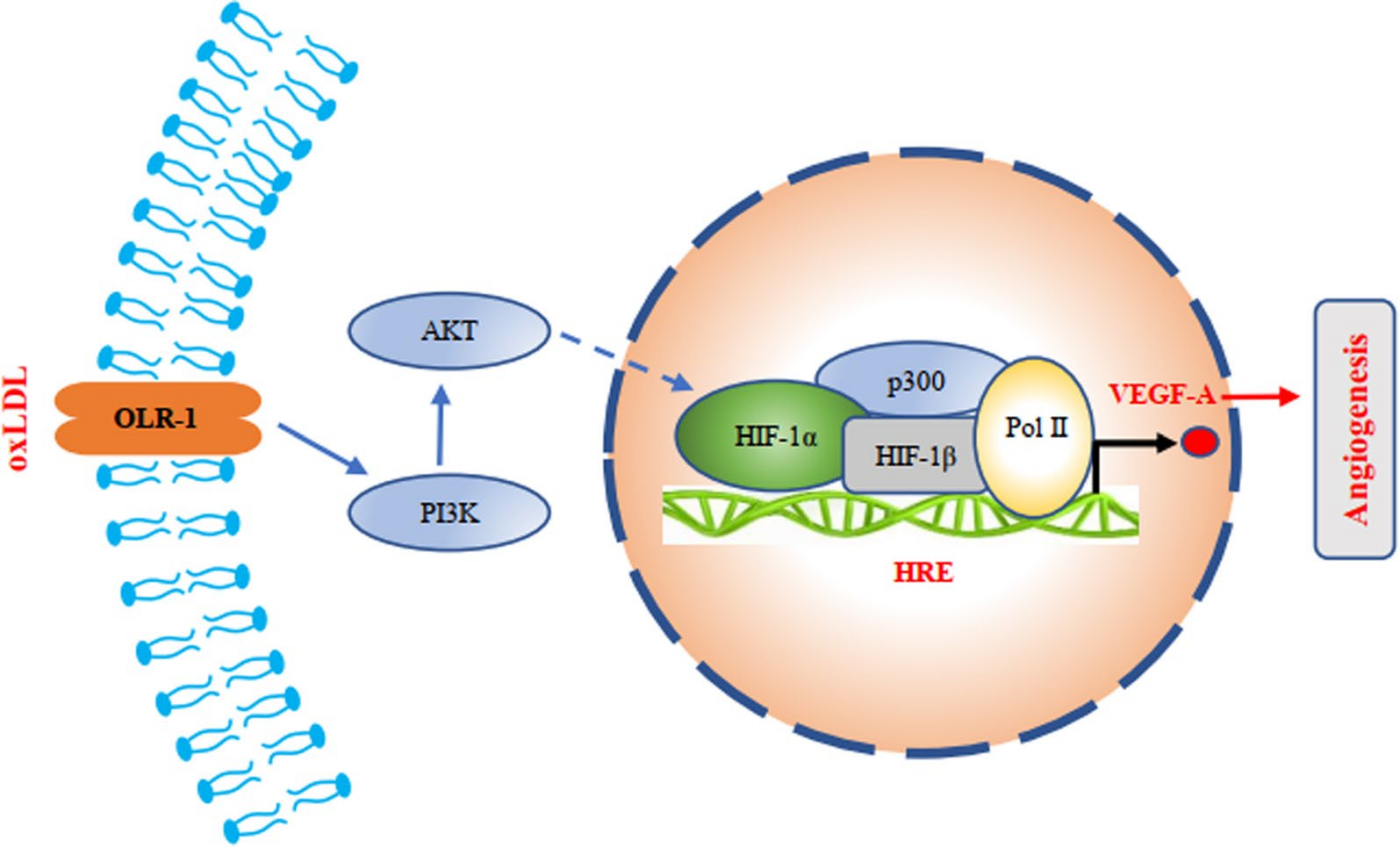
Schematic diagram of oxLDL upregulating VEGF-A expression

To explore the molecular mechanism by which oxLDL upregulates VEGF-A in hEECs, we used RNA-seq to detect changes in the RNA transcriptome in the hEECs treated with oxLDL. Sequencing results showed that the transcription level of VEGF-A was significantly upregulated. Combined with KEGG signalling pathway analysis, the results showed that the transcription level of PI3K-AKT signalling pathway-related genes changed significantly (Fig. 5), suggesting that the PI3K-AKT signalling pathway may participate in the regulation of VEGF-A expression by oxLDL. VEGF-A expression was reported to be regulated by HIF-1α, while the activity of oxLDL is often mediated by its specific receptor, OLR1. Combined with the results of RNA analysis, these findings indicated that oxLDL may enhance HIF-1α signalling activation via the OLR1-PI3K-AKT axis and then upregulate the expression of VEGF-A. To test the above scientific hypothesis, we treated hEECs with oxLDL after OLR1 receptor interference and then detected p-AKT and HIF-1α expression levels by Western blot and immunofluorescence techniques. The results showed that oxLDL could promote the phosphorylation level of AKT and then upregulate HIF-1α expression (Fig. 6A–D; Additional file 1: Fig. S1A, B, C, D). For the opposite effects, we applied AKT and HIF-1α signalling pathway inhibitors to treat hEECs before oxLDL incubation. Finally, we observed that the above effects of oxLDL were neutralized to some extent (Fig. 6E, F; Additional file 1: Fig. S1E, F), indicating that the OLR1-PI3K-AKT-HIF-1α axis indeed mediated the upregulation of VEGF-A expression by oxLDL (Fig. 7).

## Discussion

Endometriosis (EMS) is a common gynaecological disease that is difficult to cure because of its similar metastatic ability to tumours (Dahiya et al. 2021; Kralickova et al. 2014). Similarly, we can also use strategies for tumour prevention and treatment to prevent and treat EMS. Vascular metastasis is not only one of the main mechanisms of tumour metastasis but also an important pathway of EMS metastasis (Laschke and Menger 2018). Therefore, antiangiogenic strategies are an effective means to prevent the progression and metastasis of EMS. Tumour cells can promote angiogenesis by paracrine VEGF-A to support vascular metastasis (Yang et al. 2013). In this study, we found that endometrial cells have similar functions, especially endometrial epithelial cells. However, EMS is a complex disease with many influential factors. The factors driving vascular metastasis are worth further investigation. Diego Raimondo et al. demonstrated that CRP may be a moderately accurate predictive marker of postoperative complications in patients who have undergone elective laparoscopic shaving for rectosigmoid deep infiltrating endometriosis (Raimondo et al. 2022). Other scholars found that hypoxia also influenced the development and progression of endometriosis (Li et al. 2021). OxLDL is an important oxidative stress factor in the blood and is also a risk factor for many diseases, including coronary atherosclerotic heart disease and cancer (Poznyak et al. 2020; Bitorina et al. 2021). We found that oxLDL can increase the risk of EMS metastasis. EMS patients with higher plasma oxLDL concentrations have a greater risk of metastasis. Further studies found that oxLDL can upregulate VEGF-A in endometrial epithelial cells and stromal cells. VEGF-A is an important factor in promoting angiogenesis (Nagy et al. 2007), indicating that oxLDL may enhance angiogenesis by upregulating the expression and secretion of VEGF-A, thus promoting the distant migration of endometrial cells. ORL1 is a specific receptor of oxLDL that mediates most functions of oxLDL, such as NF-κB and TNF-α signalling pathway activation, promoting the formation of foam cells in the vasculature and enhancing the proliferation and metastasis of tumour cells (Pirillo et al. 2013; Ma et al. 2019; Poznyak et al. 2020; Feng et al. 2014). HIF-1α signalling pathway activation can directly initiate the transcription and expression of VEGF-A (Palazon et al. 2017). Therefore, we propose a scientific hypothesis: oxLDL upregulates the expression of VEGF-A in endometrial epithelial cells through OLR1. Finally, our experimental results verified our scientific hypothesis. Moreover, we found that oxLDL upregulated HIF-1α expression to enhance HIF-1α signalling pathway activation. How does oxLDL upregulate HIF-1α expression through OLR1? We screened the signalling pathways significantly affected by oxLDL treatment with RNA-seq. Among these pathways, the AKT signalling pathway was reported to regulate HIF-1α expression (Pez et al. 2011; Zhang et al. 2018). Through further experiments, oxLDL was shown to activate the AKT signalling pathway in endometrial epithelial cells. Moreover, the upregulation of HIF-1α by oxLDL was observed to be reduced after blocking the AKT signalling pathway, which proved that oxLDL enhanced HIF-1α through the AKT signalling pathway, promoted the activation of the HIF-1α signalling pathway, and finally upregulated the expression of VEGF-A to enhance the distant metastasis of endometrial cells.

## Conclusion

This project clarifies the molecular mechanism by which oxLDL upregulates the expression of VEGF-A in endometrial cells through the AKT-HIF-1α signalling pathway and then promotes the distant metastasis of EMS, providing new theoretical support and therapeutic targets for the prevention and treatment of EMS.

## Supplementary Information

**Additional file 1: Figure S1.** OxLDL upregulated VEGF-A expression and secretion via OLR-1/PI3K/Akt signalling. (A&B) p-AKT and HIF-1α expression in each group of hESCs treated with oxLDL (50 μg/ml) or PBS for 48 h was examined by Western blots. The histogram represents the greyscale of each lane from the Western blot. The number in each group was 3. * represents p < 0.05. (C&D) p-AKT and HIF-1α expression in the hESCs treated with oxLDL (50 μg/ml) or PBS for 48 h was examined by immunofluorescence. (E&F) VEGF-A expression in each group of hESCs treated with oxLDL (50 μg/ml) for 48 h, the AKT inhibitor perifosine (20 mM) and HIF-1α LW6 (20 mM) was examined by Western blotting. The histogram represents the greyscale of each lane from the Western blot. The number in each group was 3. * represents p < 0.05.

## Data Availability

All data generated or analyzed during this study are included in this article.

## Abbreviations

EMS: Endometriosis
MVD: Microvascular density
oxLDL: Oxidized low-density lipoprotein
OLR1: Oxidized low-density lipoprotein (lectin-like) receptor 1
hEECs: Human endometrial epithelial cells
hESCs: Human endometrial stromal cells
hUVECs: Human umbilical vein endothelial cells
VEGF-A: Vascular epithelial growth factor A
HIF-1: Hypoxia inducible factor-1
FBS: Fetal bovine serum
