# Supplementary figures and images for: Oxidized LDL promotes EMS-induced angiogenesis by increasing VEGF-A expression and secretion by endometrial cells
Source: Mol Med. 2022 Dec 12;28:151. doi: 10.1186/s10020-022-00582-6 (PMC9743733; doi:10.1186/s10020-022-00582-6)

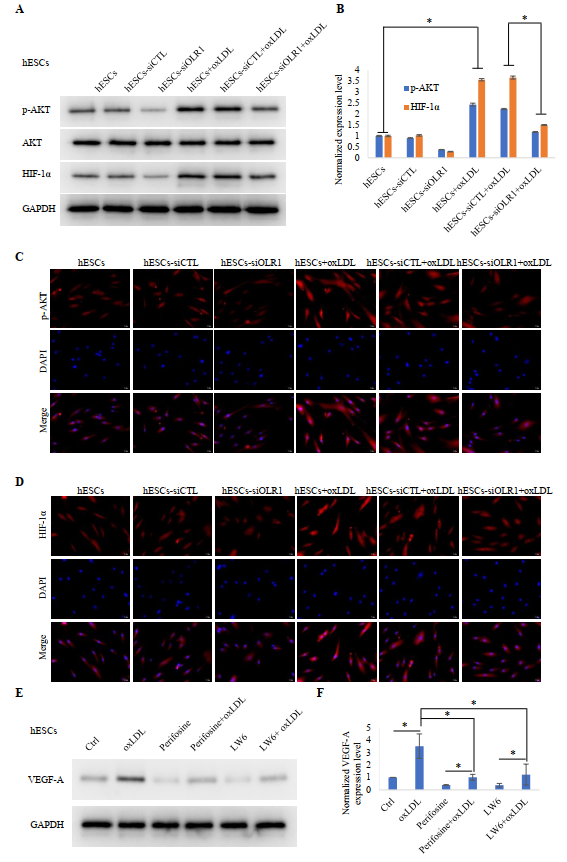

Supplement: Supplementary file 1 — Additional file 1: Figure S1. OxLDL upregulated VEGF-A expression and secretion via OLR-1/PI3K/Akt signalling. (A&B) p-AKT and HIF-1α expression in each group of hESCs treated with oxLDL (50 μg/ml) or PBS for 48 h was examined by Western blots. The histogram represents the greyscale of each lane from the Western blot. The number in each group was 3. * represents p < 0.05. (C&D) p-AKT and HIF-1α expression in the hESCs treated with oxLDL (50 μg/ml) or PBS for 48 h was examined by immunofluorescence. (E&F) VEGF-A expression in each group of hESCs treated with oxLDL (50 μg/ml) for 48 h, the AKT inhibitor perifosine (20 mM) and HIF-1α LW6 (20 mM) was examined by Western blotting. The histogram represents the greyscale of each lane from the Western blot. The number in each group was 3. * represents p < 0.05. [file 10020_2022_582_MOESM1_ESM.png]
